# Sarcoidosis presenting as massive splenomegaly and severe epistaxis, case report

**DOI:** 10.1016/j.amsu.2020.03.007

**Published:** 2020-04-08

**Authors:** Austen Stoelting, Shawn Esperti, Nino Balanchivadze, Valentino Piacentino, Andrew Mangano

**Affiliations:** a Department of Internal Medicine, Grand Strand Medical Center, 809 82nd Pkwy, Myrtle Beach, SC, 29572, USA; b Department of General Surgery, Grand Strand Medical Center, 809 82nd Pkwy, Myrtle Beach, SC, 29572, USA

**Keywords:** Sarcoidosis, Thrombocytopenia, Splenomegaly, Splenic artery aneurysm, Epistaxis

## Abstract

Sarcoidosis is a multisystem disorder of unknown etiology. Extrapulmonary sarcoidosis can involve any organ, but isolated spleen involvement is rare. Diagnosis can be challenging as other etiologies may have similar presentations. A 58-year-old African American female presented with life threatening epistaxis, anemia, refractory thrombocytopenia, and massive splenomegaly. Lymphoproliferative, infectious, and autoimmune etiologies were eliminated with laboratory testing and bone marrow biopsy. The patient had multiple splenic artery aneurysms precluding an open diagnostic splenectomy. Partial splenic artery embolization was performed, which normalized the platelet count and resolved the spontaneous bleeding. This allowed diagnostic splenectomy and splenic artery repair to be safely performed. Surgical pathology demonstrated extensive non-caseating granulomas consistent with sarcoidosis. We present this case to demonstrate the omnipotent nature of sarcoidosis and a complex multi-disciplinary approach for successful diagnosis and treatment.

## Highlights

• Sarcoidosis rarely presents with extrapulmonary involvement leading to life-threatening clinical scenarios.
• Splenic sarcoidosis as a source of life-threatening platelet sequestration.
• Splenectomy can be used as diagnostic and therapeutic intervention in sarcoidosis.
• Partial splenic artery embolization provides safe alternative to open splenic artery aneurysm repair.

## 1 Introduction

Sarcoidosis is an idiopathic multisystem disorder characterized by formation of non-caseating granulomas, with greater than 90% of cases isolated to the lungs [1,2]. Extrapulmonary involvement most commonly manifests in the skin, lymph nodes, eyes, heart, GI tract, liver, and spleen [3]. Recent data suggests that splenic involvement in sarcoidosis ranges from 10 to over 50% of cases [4]. In a review of 6074 patients with sarcoidosis, only 3% had massive splenomegaly [3]. Recently, massive splenomegaly has been defined as spleen reaching the iliac crest, crosses the midline, or weights more than 1500 g [5]. The etiology remains unknown; however, over the past decade several genomic studies have identified potential HLA and non-HLA alleles as well as a variety of infectious agents that appear to be associated with the disease [6]. HLA-DRB1*04/*15 has been associated with extrapulmonary sarcoidosis [7,8].

The potential systemic involvement of sarcoidosis can make diagnosis and recognition difficult. Diagnostic criteria includes compatible clinical and radiographic presentation, histologic evidence of non-caseating granulomas, and exclusion of other granulomatous diseases [6,7,9]. Incidence is higher in women and in the African American population. We present a case of a 58-year-old African American female who presented with life threatening epistaxis, anemia, refractory thrombocytopenia, and multiple splenic artery aneurysms to demonstrate the intricacies of sarcoidosis with a unique and multi-disciplinary approach to diagnosis and treatment.

## 2 Case report

**Fig. 1:**
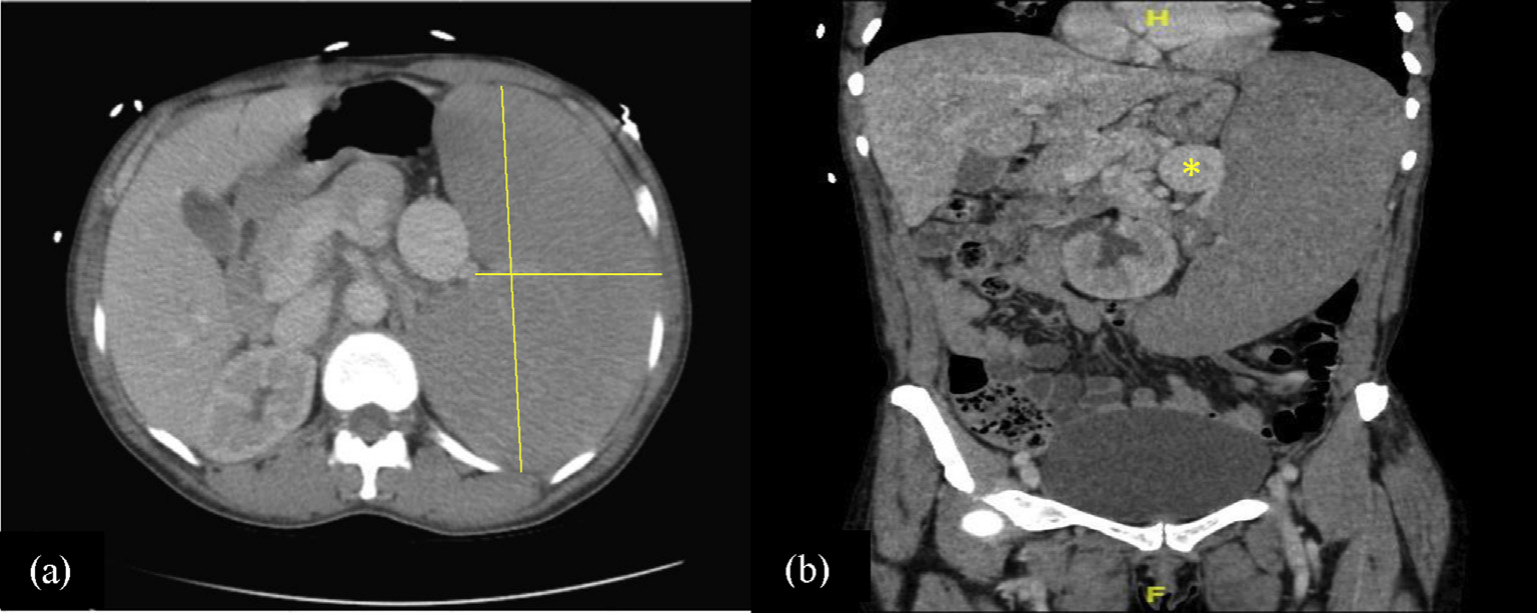
(a) CT abdomen and pelvis with contrast demonstrated marked splenomegaly, spleen measuring 25 cm craniocaudad, 8 cm transverse, 18.5 cm anterior-posterior. (b) Coronal view showing marked enlargement of the splenic veins, multiple splenic artery aneurysms and massive splenomegaly. Largest aneurysm was 6 cm.

A 58-year-old African American female with history of iron deficiency anemia, two independent episodes of unprovoked deep vein thrombosis (not on active anticoagulation), and one first trimester miscarriage presented with severe epistaxis. Review of systems was positive for nausea, vomiting, early satiety, and a 10 pound unintentional weight loss over the past year. She denied any fevers, night sweats, respiratory, or cardiac symptoms. Family history is notable for a sister with an unknown bleeding disorder. Vital signs on admission: T 97.8 °F, HR 129, BP 140/65, R 17, SpO_2_100% on room air. Physical exam was significant for massive splenomegaly extending into the pelvis, pallor, and dried blood in bilateral nares. On admission, pertinent labs included white blood cell of 6.9 K/mm3, hemoglobin of 4.8 g/dl, and platelet count of 6000 K/mm^3^. Basic metabolic panel was normal. Liver chemistry revealed a mildly elevated total bilirubin of 1.5 mg/dl with direct bilirubin of 0.4 mg/dl, aspartate aminotransferase of 10 units/L, alanine aminotransferase of 13 units/L, and alkaline phosphatase of 64 units/L. Serum albumin was low at 3.2 gm/dl. Thrombocytopenia work-up included a normal fibrinogen, PTT, PT/INR, and peripheral smear did not reveal schistocytes but demonstrated increased number of metamyelocytes. No platelet clumping was noted. Laboratory workup for hemolysis included an elevated LDH of 262 U/L (normal <200U/L), normal haptoglobin, and reticulocyte count of 9.5%. CT abdomen and pelvis without contrast was significant for massive splenomegaly with spleen measuring 25 cm craniocaudad, 8 cm transverse, and 18.5 cm anterior-posterior without discrete splenic lesions, as well as numerous splenic artery aneurysms, largest measured 6 cm. There was normal contour of the liver, arguing against underlying chronic liver disease or cirrhosis (Fig. 1a and b). CT chest was void of hilar adenopathy or pulmonary infiltrates but showed bibasilar scarring fibrosis and basilar traction bronchiectasis. There was no prior imaging for comparison and the patient denied any previous pulmonary complaints.

**Fig. 2:**
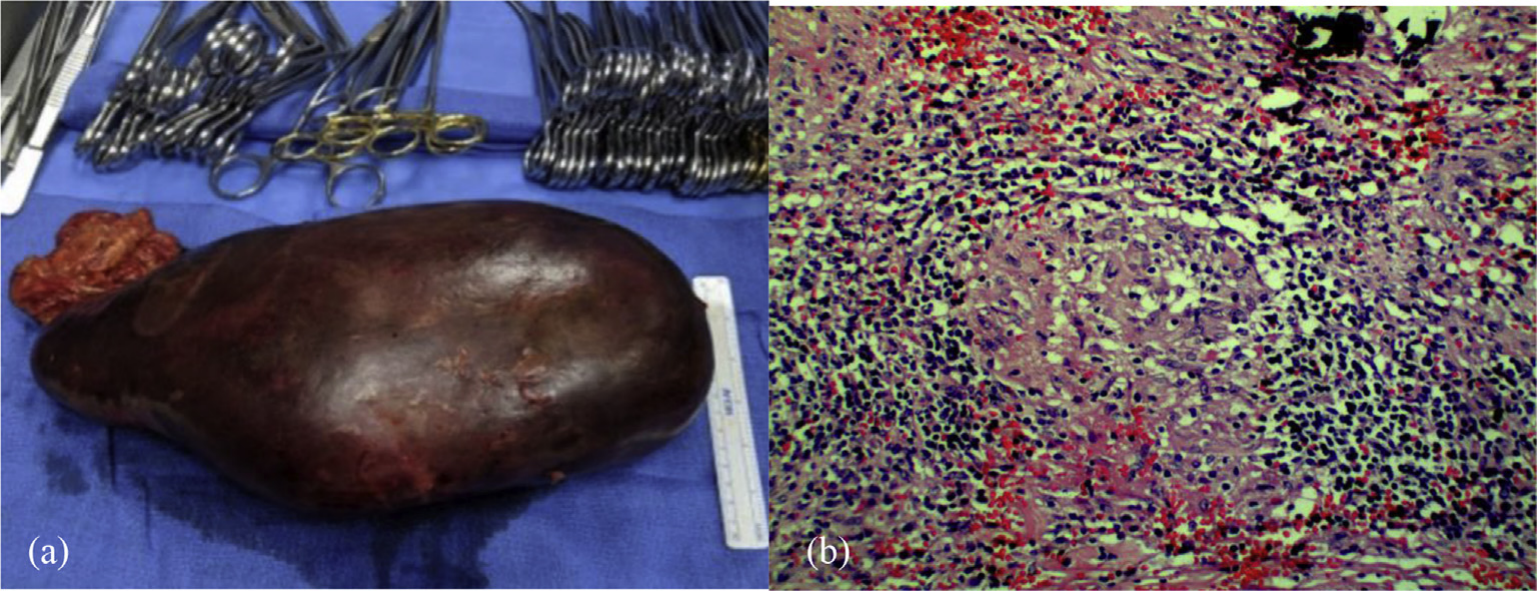
(a) Gross specimen of spleen measuring 28 × 20 × 12 cm. Weight 2875 g. (b) Microscopic examination showing a non-caseating granuloma (HEx 20).

Hematology was consulted and multiple differential diagnoses were considered, including lymphoproliferative disorders and infiltrative disorders such as sarcoidosis. Imaging of chest, abdomen, and pelvis did not reveal lymphadenopathy or any other masses that could be biopsied for definitive diagnosis. Bone marrow aspirate and core biopsy evaluation was not suggestive of marrow failure syndromes, lymphoproliferative etiologies, or malignancy. Infectious and autoimmune etiologies were ruled out. Multiple platelet transfusions did not improve the platelet count and she continued to experience severe epistaxis requiring multiple packed red blood cell transfusions. Diagnostic splenectomy was required to distinguish between sarcoidosis and lymphoproliferative disorders. Given the size of the spleen, bleeding, and severe thrombocytopenia, surgical splenectomy was deemed unsafe. In addition, multiple splenic artery aneurysms and size of the largest aneurysms limited endovascular approach. Interventional Radiology performed a partial splenic artery embolization, which normalized the platelet count from 2 to 154 K/mm^3^ within three days. Spontaneous bleeding resolved after partial splenic artery embolization, which allowed Vascular Surgery to perform a diagnostic splenectomy and safe repair of the splenic artery aneurysms (Fig. 2a). Surgical pathology demonstrated extensive non-caseating granulomas consistent with sarcoidosis (Fig. 2b).

## 3 Discussion

The liver and spleen are the primary organs involved in extrapulmonary manifestations of sarcoidosis. In the spleen, massive splenomegaly is the most common presentation, followed by multiple splenic lesions [3]. This case of massive splenomegaly was confounded by both splenic sarcoidosis and multiple splenic artery aneurysms leading to aggressive platelet sequestration. Despite consecutive platelet transfusions, platelet counts remained under 10 thousand, and hemostasis was unable to be achieved, resulting in life-threatening epistaxis. Splenectomy in sarcoidosis is indicated for patients who fail to respond to pharmacotherapy, massive splenomegaly with pressure-related symptoms, prophylaxis for splenic rupture, treatment for refractory cytopenias, or diagnostic exclusion of malignancy [3].

Splenic artery aneurysms are the most common visceral artery aneurysms, but they are quite rare, with a prevalence of less than 1% [10]. Often patients present with nonspecific symptoms such as diffuse, dull abdominal pain, or left upper quadrant pain. In some cases, patients can present in hemorrhagic shock secondary to rupture [11]. Aneurysm size greater than 2 cm poses the highest risk for rupture and should be treated even in asymptomatic individuals [10]. Reported cases have been identified in patients with hypertension, portal hypertension, cirrhosis, liver transplantation, pregnancy, and less commonly infiltrative, inflammatory, or infectious etiologies [13]. In these conditions, increased blood flow through the splenic artery is the likely cause of the aneurysm. This can lead to irreversible damage of the tunica media resulting in aneurysm development [12]. Moderate splenomegaly has been associated with up to 50% of splenic artery aneurysms [13].

Gold standard treatment for splenic artery aneurysms is open repair; however, the endovascular approach has been an emerging option [14]. A 2014 systematic review and meta-analysis comparing the three major treatment modalities (open, endovascular, and conservative) for splenic artery aneurysm repair found that endovascular repair has significantly lower perioperative mortality but higher re-intervention rates [14]. Open repair was associated with a higher 30-day mortality but fewer late complications and re-interventions [14]. In this case, having multiple splenic artery aneurysms with largest aneurysm of 6 cm limited the ability to perform endovascular repair. Partial splenic artery embolization decreased platelet sequestration, improved platelet counts, and allowed for safe, open repair of splenic artery aneurysms while salvaging viable splenic tissue to help establish a histologic diagnosis for our patient.

Perioperative management in patients requiring splenectomy is critical to reduce mortality from the procedure. Prior to splenectomy, there are several preoperative considerations that should be taken. First, all alternatives to splenectomy should be investigated prior to proceeding with invasive surgery. Non-invasive testing such as CT scans looking for alternative lesions to biopsy or bone marrow biopsy with flow cytometry should be considered [15]. Isolated splenic biopsy of focal lesions can result in positive diagnosis without need for splenectomy [16]. Preoperative testing should include CBC, PT, PTT, SPEP, and bone marrow biopsy. The optimal platelet count prior to major surgery should remain above 50,000 K/mm^3^ [17]. Preoperative splenic artery embolization has been successfully utilized to reduce the risk of hemorrhage during open splenectomy [18,19]. The spleen is an important organ for host defense against encapsulated organisms. Vaccines should be administered at least two weeks prior to elective splenectomy, with ideal timing 10–12 weeks prior to splenectomy. Emergent cases should receive vaccines 14 days after splenectomy [20]. Current CDC recommendations include pneumococcal vaccinations with PCV13 and PPSV23, Haemophilus *influenzae* type B, meningococcal vaccines with MenACWY and MenB, and annual seasonal flu vaccine. In addition to developing life-threatening infection postoperatively, patients are at an increased risk of vascular complications including pulmonary embolism, deep vein thrombosis, portal and splenic vein thrombosis [21]. Standard venous thromboembolism prophylaxis should be utilized.

## 4 Conclusion

This report illustrates an unusual case of bicytopenia, massive splenomegaly, and life-threatening epistaxis caused by a combination of splenic sarcoidosis and multiple splenic artery aneurysms. In patients presenting with massive splenomegaly and thrombocytopenia, splenic sarcoidosis should be considered in the differential along with other causes of splenic sequestration including infection, autoimmune cytopenias, leukemia, lymphoma, liver disease, vascular obstruction, and extramedullary hematopoiesis. Partial splenic artery embolization may be a reasonable approach to decrease splenic sequestration and allow for safe surgical intervention when pathologic diagnosis is needed. Multidisciplinary approach among internists, hematologists, surgeons, and interventional radiologists is necessary for successful diagnosis and treatment.

## Ethical approval

This study was approved by Ethics Committee.

## Sources of funding

This study has not received any funding.

## Author contribution

Study concept or design – AS, SE, NB, AM.

Data collection – AS, AM.

Data interpretation – AS, SE, NB, AM.

Literature review – AS, AM.

Drafting of the paper – AS, NB, AM.

Editing of the paper – AS, SE, NB, AM, VP.

## Trial registry number

1. Name of the registry: Not applicable
2. Unique Identifying number or registration ID:
3. Hyperlink to your specific registration (must be publicly accessible and will be checked):

## Guarantor

Austen Stoelting, M.D.

## Consent

The patient provided informed written consent prior to submission of this manuscript.

## Provenance and peer review

Not commissioned, externally peer reviewed.

## Disclaimer

This research was supported (in whole or in part) by HCA and/or an HCA affiliated entity. The views expressed in this publication represent those of the author(s) and do not necessarily represent the official views of HCA or any of its affiliated entities.

## Declaration of competing interest

None.
